# Identification and Analysis of Rice Yield-Related Candidate Genes by Walking on the Functional Network

**DOI:** 10.3389/fpls.2018.01685

**Published:** 2018-11-20

**Authors:** Jing Jiang, Fei Xing, Chunyu Wang, Xiangxiang Zeng

**Affiliations:** ^1^School of Aerospace Engineering, Xiamen University, Xiamen, China; ^2^School of Computer Science and Technology, Harbin Institute of Technology, Harbin, China; ^3^School of Information Science and Engineering, Xiamen University, Xiamen, China

**Keywords:** rice, yield, random walking, function, network

## Abstract

Rice (*Oryza sativa* L.) is one of the most important staple foods in the world. It is possible to identify candidate genes associated with rice yield using the model of random walk with restart on a functional similarity network. We demonstrated the high performance of this approach by a five-fold cross-validation experiment, as well as the robustness of the parameter r. We also assessed the strength of associations between known seeds and candidate genes in the light of the results scores. The candidates ranking at the top of the results list were considered to be the most relevant rice yield-related genes. This study provides a valuable alternative for rice breeding and biology research. The relevant dataset and script can be downloaded at the website: http://lab.malab.cn/jj/rice.htm.

## Introduction

Rice (*Oryza sativa* L.) is one of the most important food crops worldwide, being used as the main food source by more than half of the global population (Mahender et al., 2016; Li et al., 2017). In the developing world, rice provides 27% of dietary energy and 20% of dietary protein (Huang et al., 2013). However, despite genetic improvements in grain yield delivered by the exploitation of semi-dwarfism and heterosis over the past 50 years, a substantial increase in grain productivity of the major crops is still required to feed a growing world population (Abe et al., 2018). The prime breeding target is to increase both grain size and grain number, because they impact both on yield potential and its end-use quality (Okada et al., 2018). However, the simultaneous improvement of grain quality and grain yield is a major challenge because of the well-established negative correlation between these two traits which is controlled by quantitative trait loci and influenced by environmental changes. Additionally, determining which genes in quantitative trait loci regulate grain size and number has not been clarified (Borzee et al., 2018; Li et al., 2018). Therefore, the identification genetic variants associated with improvements in grain yield would facilitate the breeding of new high-yielding rice varieties and may also be applicable to other crops (You et al., 2017).

Vast numbers of genetic variants have been detected by traditional genome-wide association studies and recent sequencing studies, and connecting the functional implications of these results to known genes has become a standard task (Li et al., 2015; Dehury et al., 2017; Torres and Henry, 2018; Wu et al., 2018). We previously developed a database, RicyerDB, to collect all known rice yield-related genes by integrating multiple omics data, information from the literature, and associated databases (Jiang et al., 2018). This work also established a search tool to query a particular gene, and to provide insights into gene functions and locations. Any rice yield-related gene can therefore be easily queried and the findings downloaded through the webpage, while candidate genes can be screened and prioritized to identify those most likely to be associated with known genes.

To achieve this goal, several approaches have been proposed from the perspective of computational systems biology (Behroozi-Khazaei and Nasirahmadi, 2017; He et al., 2017; Liu E. et al., 2017; Liu Y. et al., 2017; Xiong et al., 2017; Maione and Barbosa, 2018; Zhang M. et al., 2018; Zhou et al., 2018). For example, the Endeavor tool uses the guilt-by-association principle to rank candidate genes according to their functional similarities to a set of predefined seed genes (Aerts et al., 2006; Tranchevent et al., 2008, 2016). In recent years, a protein–protein interaction (PPI) network has been developed to achieve a global inference of entire genes (Liu et al., 2010; Lee, 2011; Rezadoost et al., 2016; Wang et al., 2016; Zeng et al., 2016; Luo and Liu, 2017; Holland and Johnson, 2018; Vlaic et al., 2018). PPI networks have also been used to provide a simplified yet systematic measure of functional similarities between genes (Chen et al., 2017a, 2018a).

Some methods for identifying yield-related genes have linked profile and sequence technology to facilitate the prediction of related genes. For example, Odilbekov et al. (2018) used machine learning and integrated this analysis with data obtained from spectroradiometer, infrared thermometer, and chlorophyll fluorescence measurements to identify the most predictive proxy measurements for studying Septoria tritici blotch disease of wheat.

Hybrid breeding is an effective tool to improve yield in rice, although parental selection remains a difficult issue. Xu et al. (2018) compared six genomic selection methods, such as least absolute shrinkage and selection operation and support vector machine, to evaluate predictabilities for different methods, and demonstrated their implementation to predict the hybrid performance of rice. Although good results have been achieved by these studies, the techniques of microarray and sequencing are nevertheless expensive.

The main target of this research was to use current knowledge to identify rice yield-related genes with network prediction methods. We proposed a computational systems biology approach for the identification of candidate genes via a random walk model on a PPI network with functional similarities (Kohler et al., 2008). Starting from known nodes, our method simulates the process in which a random walker travels to its neighbors or jumps to itself in the network, scores a gene using the probability that the walker stays in the gene at a steady state, and then ranks candidate genes according to their scores. Using a series of cross-validation experiments, we systematically demonstrated the robustness of our method, and applied our approach to predict a landscape of associations between known genes and candidates.

## Materials and Methods

### Flowchart Overview

**FIGURE 1:**
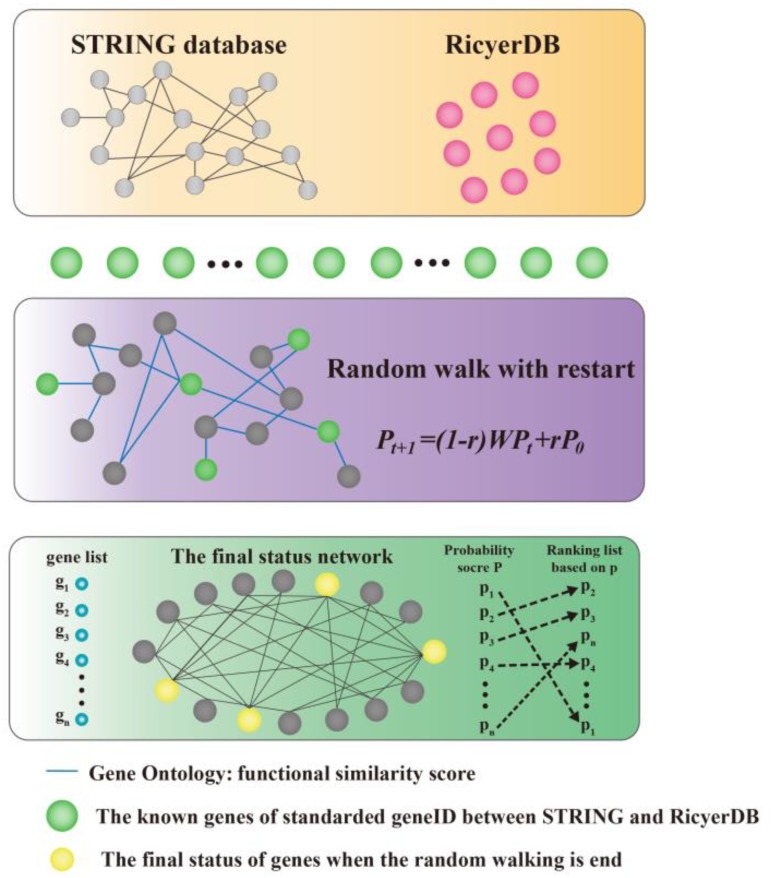
Illustration of the proposed method. Our method takes a set of seed genes as the input, and gives a ranking list of the candidates as the output. A functional similarity network was constructed by applying a random walk with restart algorithm to the network to obtain scores for candidate genes, and then the candidates were ranked according to their scores.

We modeled the problem of identifying candidate genes associated with a set of known genes as a prioritization problem, and proposed to solve this problem using a three-step approach. As shown in Figure 1, taking the set of known genes as input, we first standardized the genes between STRING (Szklarczyk et al., 2015) and RicyerDB (Jiang et al., 2018). Then, we constructed a protein–protein network that scores the edges through functional similarities. This procedure applied a RWR algorithm to the network to calculate a score for each candidate gene, and then ranked the candidates to obtain a ranking list as the output (Chen et al., 2012a,b; Chen, 2016; Chen X. et al., 2016; Li et al., 2016; Peng et al., 2016; Zhu et al., 2018). Finally, the top candidate gene was verified according to its function and by the published literature.

### Construction of the Functional Similarity Network

The functional similarity network is described as a graph G = (V, E), where V represents the nodes of the network and E stands for the edges of the network. The background network comes from the STRING database because of existing potential associated interactions among the proteins. The known rice yield-related genes were identified from our previous work with RicyerDB (Jiang et al., 2018). To standardize gene names between STRING and RicyerDB, genes were retrieved by reference to National Center for Biotechnology Information gene names. Functional similarities among genes in the background network were considered by scoring E for GO annotations. Using the latest release of the GO database (Ashburner et al., 2000; Chen L. et al., 2016; Raza, 2016; The Gene Ontology, 2017), edges were scored for a shared functional significance score of genes in the network that were annotated with GO terms.

The shared functional significance score *F*(i,j) between gene i and j was measured by the Weighted Shared Functions approach, which considered a gene’s functions as a set of functional categories in GO. The functions shared by a small number of genes are taken to be far more significant than ones shared by a large number of genes. Each function had its own significance, which was defined as the inverse number of genes sharing the function. When two genes, i and j, have m functions in common, i.e., *F*(i)∩*F*(j) = (f_1_, f_2_, …, f_*m*_), *F*(i,j) was given as the total sum of the significance of the functions shared between them as follows:

$$F(i,j)=\sum_{n=1}^{m}\mathrm{sig}(f_{n})$$

$$\mathrm{sig}(f_{n})=\frac{1}{\left|\mathrm{Gene}(f_{n})\right|}$$

Here sig(f*_n_*) denotes the significance of a function f*_n_*(*n* = 1,2,..., m) shared between genes i and j, | Genes (f*_n_*)| is the number of genes sharing a function f_*n*_. We calculated the ranking score, p, for each gene in the disease-related network and ranked these genes in the descending order of p.

### Random Walking on the Functional Similarity Network

We achieved the goal of identifying candidates related to known seeds by calculating a score for each candidate and then ranking the candidates to obtain a ranking list. The higher the rank, the more likely the gene was to be related to the given source nodes. For this purpose, we adapted the RWR method in the functional similarity network.

At the beginning, the walker chooses the seeds as the starting point. In each step of the walking process, the walker may start on a new journey with probability r or move on with probability 1−*r*. When moving on, the walker may move at random to one of its direct neighbors.

In our application, the initial probability vector P_0_ was constructed such that equal probabilities were assigned to the nodes representing members of the disease, with the sum of the probabilities equal to 1. This is equivalent to letting the random walker begin from each of the known disease genes with equal probability. The transition matrix W is the column-normalized adjacency matrix of the graph, and P_*t*_ is a vector in which the ith element holds the probability of being at node i at time step t. Formally, the RWR is defined as:

$$P_{t+1}=(1-r){\mathrm{WP}}_{t}+{\mathrm{rP}}_{0}$$

Candidate genes were ranked according to the values in the steady-state probability vector P. P vector changes with time t, while it is possible to obtain it by explicitly calculating Equation (1) until convergence. The iteration is finished when the change between P_*t*_ and P_*t*+1_ falls below 10^−10^. In this paper, we set default values for parameters *r* = 0.3 (see Results section for details).

### Validation Method

We adopted a five-fold cross-validation experiment to assess the capability of RWR to identify the left seeds. All seed genes were divided equally into five parts, then one part was removed as a test set, and added to the candidate genes. All candidate genes were ranked by RWR to determine the ranking of the test gene. This procedure was repeated until all seed genes were used up as test genes.

In the context of the functional similarity network, the above validation procedure was equivalent to removing one part of the seed genes to candidate genes and determining whether candidates containing these seeds could receive a high rank. The r parameter of RWR ranged from [0,1] and was used to identify the ranking of the five parts. ROC curves were plotted, and areas under the ROC curve (AUC) values were used to evaluate the performance of r.

## Results

### Data Sources

We obtained the rice background protein–protein network from the STRING database. In the network, protein associations were either directly derived from physical interactions or functional links from experimental evidence and computational methods (Jensen et al., 2009). The network composes of 6561 nodes and 567034 edges, which represent proteins and interactions between them, respectively. In our study, 136 known genes were selected as seed genes and other genes as candidate genes. We downloaded *O. sativa* Japonica protein network data through STRING version 10.5 (Szklarczyk et al., 2015).

Proteins with accurate functional annotations are vital to biological research. We obtained functional annotation information from the GO Consortium (Ashburner et al., 2000), and downloaded GO annotations of *O. sativa* from the most recent GO version. GO enrichment analysis is used to interpret high-throughput molecular data. GO annotation is the list of all annotated genes linked to ontological terms describing those genes.

The RicyerDB database integrates publicly available resources to construct a public platform for browsing and the interactive visualization of yield-related genes. The first release of RicyerDB contained more than 400 manually curated gene information entries which were all associated with rice yield.

### Performance of the Proposed Method

The score vector P (the probability of being at the current node) for all genes in the network was calculated based on the ranking of corresponding *r* coefficients. Candidate genes were then ranked in the descending order of P score.

**FIGURE 2:**
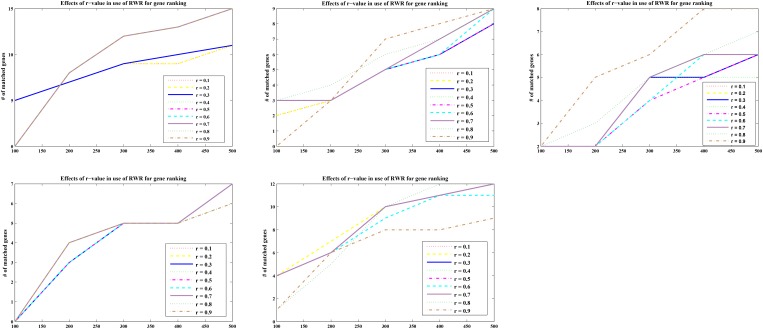
Five-fold cross validation of the parameter r in RWR. The abscissa represents the top 500 ranking positions, and the ordinate represents the number of matching seed nodes.

For optimal parameters, genes were also ranked according to the calculated p scores with nine different *r*-values (*r* = 0.1, 0.2, 0.3, 0.4, 0.5, 0.6, 0.7, 0.8, and 0.9). The matching numbers of the five-part seed genes were applied to assess the effectiveness of RWR. In Figure 2 listed the five cases of all, the number of matched seeds among the top 500 (every 100 is a measurement cutoff) in the ranking list of *r* = 0.3 was higher than other *r*-values in most cases.

**FIGURE 3:**
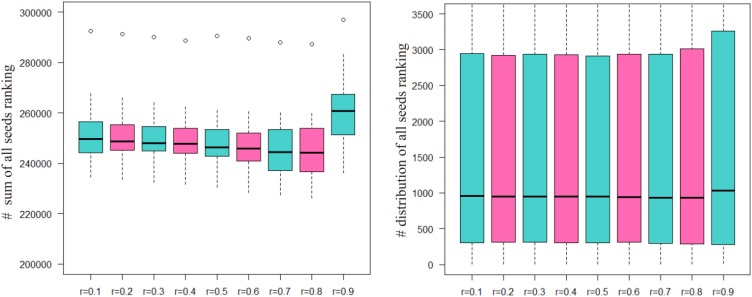
The ranking of all seeds in different *r*-values after 100 times fivefold cross validation. The number of ordinate in left part presents the sum of all seeds ranking and in right part presents the ranking position of all seeds.

The sum of the numbers of matched seed nodes in all ranking results was determined, and *r* = 0.3 was shown to have the maximum match in general. Finally, the parameter *r* = 0.3 was selected to calculate vector P to obtain the ranking results. Further to detect the robustness of parameter r, we repeated the five-fold cross validation 100 times. Then we applying statistical analysis to compare the ranking of all seeds at different *r*-values in our model, the results were shown as Figure 3.

### Prioritization of Candidate Genes and Validation by Literature Review

In the functional similarity network, all candidate genes were prioritized by RWR according to vector P at the final status. We manually searched the 100 top candidate genes (Table 1) in PubMed^1^ for their association with yield. This verified eight candidate genes associated with rice production. The LOC_Os11g40150 (rank 39) alias is OsRad51A1, which is a key component of homologous recombination in DNA repair. Direct interaction with OsNAC14 recruits factors involved in DNA damage repair and defense response, resulting in an improved tolerance to drought (Shim et al., 2018). LOC_Os04g37619 (rank 11) named ZEP, which is one of the key genes that involved hormone abscisic acid biosynthesis in rice by ion beam. Irritation can enhance the expression of genes involved in ABA biosynthesis, resulting in increasing content of endogenous plant hormone abscisic acid in rice (Chen et al., 2014).

**Table 1:** The top 100 candidate genes in the ranking list.

Ranking	Gene name	P score	PubMedID
1	LOC_Os06g09390	0.001487617	PMID: 20713616, PMID: 27555860
2	LOC_Os06g50480	0.001475286	
3	LOC_Os02g02480	0.00146746	
4	LOC_Os08g42470	0.001461294	
5	LOC_Os01g03340	0.000941415	
6	LOC_Os01g03390	0.00080268	PMID: 12972663
7	LOC_Os01g04040	0.00080268	
8	LOC_Os01g04050	0.00080268	
9	LOC_Os07g02350	0.000775571	PMID: 16240106, PMID: 11416158
10	LOC_Os08g02640	0.000669873	
11	LOC_Os04g37619	0.000640376	PMID: 24634194
12	LOC_Os11g35500	0.00062345	PMID:29813124, PMID:29402905
13	LOC_Os05g41970	0.000594578	PMID: 1731968
14	LOC_Os12g16890	0.000594578	
15	LOC_Os01g03680	0.000584668	
16	LOC_Os07g10580	0.000564849	PMID: 28158863, PMID: 22108719
17	LOC_Os06g50340	0.000561268	PMID: 19704753, PMID: 16511358
18	LOC_Os10g14150	0.000555163	PMID: 19201764
19	LOC_Os01g55540	0.000551598	PMID: 15753104
20	LOC_Os10g22860	0.00054974	PMID: 23384860, PMID: 28101092
21	LOC_Os10g32990	0.000547737	PMID: 23384860, PMID: 28101092
22	LOC_Osm1g00450	0.000540982	
23	LOC_Os01g60670	0.000536737	
24	LOC_Os07g11410	0.00053512	
25	LOC_Os01g13800	0.000533159	
26	LOC_Os02g13780	0.000533159	
27	LOC_Os10g06760	0.000533159	PMID: 23384860, PMID: 28101092
28	LOC_Os10g13970	0.000533159	PMID: 23384860, PMID: 28101092
29	LOC_Os10g19160	0.000533159	PMID: 23384860, PMID: 28101092
30	LOC_Os02g57530	0.000532385	PMID: 14754915
31	LOC_Os10g21810	0.000529529	
32	LOC_Os01g47730	0.000507068	
33	LOC_Os07g11920	0.000505391	PMID: 28158863, PMID: 22108719
34	LOC_Os01g07870	0.00049357	
35	LOC_Os03g54790	0.000492652	
36	LOC_Os01g18670	0.000492651	
37	LOC_Os07g42300	0.000483507	PMID: 24466124
38	LOC_Os11g10100	0.000478643	
39	LOC_Os11g40150	0.000478361	PMID:28071676
40	LOC_Os12g31370	0.000478361	PMID:28071676
41	LOC_Os03g05740	0.000472443	
42	LOC_Os08g38720	0.000468006	
43	LOC_Os03g50330	0.000462237	
44	LOC_Os04g08740	0.000461766	PMID: 19417056
45	LOC_Os01g42650	0.000461755	PMID: 16263700
46	LOC_Os03g27290	0.000460621	PMID: 19217306, PMID: 15672456
47	LOC_Os10g39670	0.000460227	
48	LOC_Os01g65230	0.000459159	
49	LOC_Os03g54780	0.000456546	
50	LOC_Os08g03640	0.000456163	
51	LOC_Os01g14830	0.000454589	
52	LOC_Os01g10820	0.000453601	
53	LOC_Os10g42110	0.000449388	
54	LOC_Os03g26860	0.000448345	
55	LOC_Os07g41750	0.000448221	
56	LOC_Os03g17580	0.000448145	
57	LOC_Os10g42940	0.000447386	PMID: 24715026, PMID: 10873582
58	LOC_Os03g03570	0.000446501	PMID: 10364408
59	LOC_Os12g43550	0.000445728	
60	LOC_Os03g49500	0.000444206	PMID: 29767552
61	LOC_Os10g04674	0.000442469	PMID: 24145853, PMID: 17986178
62	LOC_Os10g06740	0.000442469	PMID: 28154240
63	LOC_Os01g05980	0.000442411	
64	LOC_Os10g33650	0.000440094	
65	LOC_Os01g18150	0.000438562	
66	LOC_Os01g22490	0.000436139	
67	LOC_Os02g18550	0.000436139	
91	LOC_Os05g50930	0.000408491	
92	LOC_Os10g39440	0.000408336	PMID: 24372780, PMID: 18335199
93	LOC_Os08g06630	0.000407594	
94	LOC_Osp1g00820	0.000407028	PMID:25658309
95	LOC_Osp1g01050	0.000407028	PMID:25658309
96	LOC_Osp1g00420	0.00040642	PMID:25658309
97	LOC_Os05g49320	0.000404017	
98	LOC_Os12g07720	0.000400566	PMID: 14756303
99	LOC_Os10g06930	0.000399998	PMID: 29356995
100	LOC_Os03g06410	0.000399411	PMID: 1731968

Taken together, of the top 100 candidate genes in the ranking list, 46 candidate genes predicted by our method had been confirmed to be correlated with rice yield in PubMed literature (Table 1). Top-ranked candidates were found to have a high confirmation rate in terms of their association with rice yield, especially top 20 candidates (Table 2).

**Table 2:** The confirmation rate of top 100 candidate genes in the ranking list.

Top *n*	Confirmation Number	Confirmation Rate
20	11	55%
30	16	53.33%
40	20	50%
60	26	43.33%
70	30	42.86%
		
80	33	41.25%
100	46	46%

*The confirmation rate was calculated by dividing the confirmation number by the corresponding number of top n. It represented the effectiveness of the confirmation.*

**FIGURE 4:**
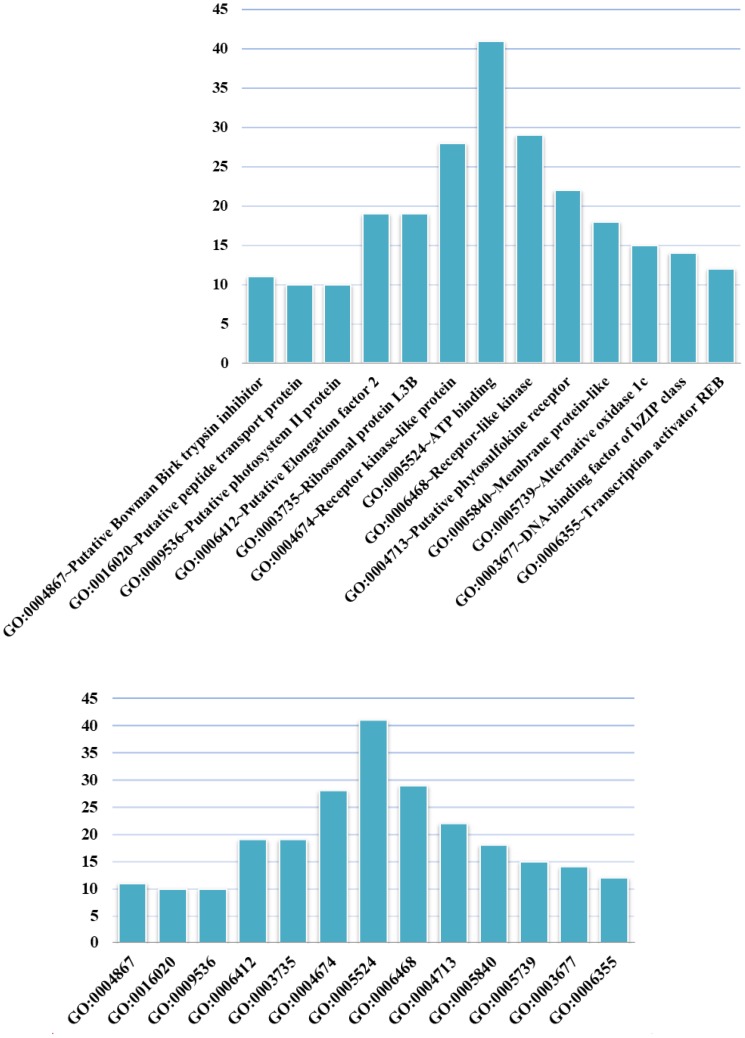
GO terms in which the top 100 candidate genes are enriched. The abscissa shows GO terms, and the ordinate represents the number of GO terms.

We conducted GO analysis to assess the functional enrichment of the top 100 candidate genes (Figure 4). The GO term having the most candidates annotated to was GO: 0005524 ∼ ATP binding, which is a binding motif within the primary structure of an ATP binding protein. A recently identified rice ATP binding cassette plays multiple roles in plant growth, development and environmental stress responses (Zhang X.D. et al., 2018). ATP binding has also been shown to play an important role in rice development (Coneva et al., 2014; Zhao et al., 2015; Chang et al., 2016; Lei et al., 2018).

## Discussion

In the present study, we identified genes associated with rice yield using the RWR method on a functional similarity network. We demonstrated the high performance of the RWR approach via a five-fold cross-validation experiment and showed the robustness of the parameter r. As an application of the RWR approach, we predicted a landscape of associations between known seeds and candidate genes.

Our work has the following advantages. First, the RWR method can predict associations among known seed genes and candidate genes with the ability to spread the information that known seeds carried via their neighbors. Second, the interaction network provides a systematic view of functional similarities between genes by calculating GO terms. Finally, the robustness of the parameter r leads to a high level of accuracy in making predictions, and the method that achieving parameter can be adapted to other dataset.

Rice is the most important food crop worldwide. Use of the RWR method in the function similarity network can identify candidate genes associated with known rice yield-related genes, while gene ranking saves experimental time in the exploitation of rice as a major crop. Future development of our research will include the collection of more rice yield-related genes via online databases and the analysis of literature. Subsequent accurate analysis involving an effective prediction algorithm will enable the prediction of novel genes that can boost rice yield. In the future, we would further develop computational models for the identification and analysis of rice yield-related microRNAs/Long non-coding RNAs based on Chen et al.’s researches (Chen and Yan, 2013; Chen and Huang, 2017; Chen et al., 2017b, 2018b).

## Author Contributions

CW designed the research. XZ performed the research. FX analyzed the data. JJ wrote the manuscript. All authors read and approved the manuscript.

## Conflict of Interest Statement

The authors declare that the research was conducted in the absence of any commercial or financial relationships that could be construed as a potential conflict of interest.

## Footnotes

1 http://www.ncbi.nlm.nih.gov/pubmed

## Abbreviations

AUC: area under the ROC curve
GO: gene ontology
ROC: receiver operating characteristic
RWR: random walk with restart.

## References

[B1] Abe K. Oshima M. Akasaka M. Konagaya K. I. Nanasato Y. Okuzaki A. (2018). Development and characterization of transgenic dominant male sterile rice toward an outcross-based breeding system. *Breed. Sci.* 68 248–257. 10.1270/jsbbs.17090 29875609 PMC5982183

[B2] Aerts S. Lambrechts D. Maity S. Van Loo P. Coessens B. De Smet F. (2006). Gene prioritization through genomic data fusion. *Nat. Biotechnol.* 24 537–544. 10.1038/nbt1203 16680138

[B3] Ashburner M. Ball C. A. Blake J. A. Botstein D. Butler H. Cherry J. M. (2000). Gene ontology: tool for the unification of biology. Gene Ontology Consortium. *Nat. Genet.* 25 25–29. 10.1038/75556 10802651 PMC3037419

[B4] Behroozi-Khazaei N. Nasirahmadi A. (2017). A neural network based model to analyze rice parboiling process with small dataset. *J. Food Sci. Technol.* 54 2562–2569. 10.1007/s13197-017-2701-x 28740314 PMC5502052

[B5] Borzee A. Heo K. Jang Y. (2018). Relationship between agro-environmental variables and breeding Hylids in rice paddies. *Sci. Rep.* 8:8049. 10.1038/s41598-018-26222-w 29795247 PMC5966434

[B6] Chang Z. Chen Z. Yan W. Xie G. Lu J. Wang N. (2016). An ABC transporter, OsABCG26, is required for anther cuticle and pollen exine formation and pollen-pistil interactions in rice. *Plant Sci.* 253 21–30. 10.1016/j.plantsci.2016.09.006 27968990

[B7] Chen L. Zhang Y.-H. Zou Q. placeChu C. Ji Z. (2016). Analysis of the chemical toxicity effects using the enrichment of Gene Ontology terms and KEGG pathways. *Biochim. Biophys. Acta Gen. Sub.* 1860 2619–2626. 10.1016/j.bbagen.2016.05.015 27208425

[B8] Chen X. You Z. H. Yan G. Y. Gong D. W. (2016). IRWRLDA: improved random walk with restart for lncRNA-disease association prediction. *Oncotarget* 7 57919–57931. 10.18632/oncotarget.11141 27517318 PMC5295400

[B9] Chen Q. F. Ya H. Y. Feng Y. R. Jiao Z. (2014). Expression of the key genes involved in ABA biosynthesis in rice implanted by ion beam. *Appl. Biochem. Biotechnol.* 173 239–247. 10.1007/s12010-014-0837-y 24634194

[B10] Chen X. (2016). miREFRWR: a novel disease-related microRNA-environmental factor interactions prediction method. *Mol. Biosyst.* 12 624–633. 10.1039/c5mb00697j 26689259

[B11] Chen X. Huang L. (2017). LRSSLMDA: laplacian regularized sparse subspace learning for MiRNA-disease association prediction. *PLoS Comput. Biol.* 13:e1005912. 10.1371/journal.pcbi.1005912 29253885 PMC5749861

[B12] Chen X. Huang L. Xie D. Zhao Q. (2018a). EGBMMDA: extreme gradient boosting machine for MiRNA-disease association prediction. *Cell Death Dis.* 9:3. 10.1038/s41419-017-0003-x 29305594 PMC5849212

[B13] Chen X. Xie D. Wang L. Zhao Q. You Z. H. Liu H. (2018b). BNPMDA: bipartite network projection for MiRNA-disease association prediction. *Bioinformatics* 34 3178–3186. 10.1093/bioinformatics/bty333 29701758

[B14] Chen X. Liu M. X. Yan G. Y. (2012a). Drug-target interaction prediction by random walk on the heterogeneous network. *Mol. Biosyst.* 8 1970–1978. 10.1039/c2mb00002d 22538619

[B15] Chen X. Liu M. X. Yan G. Y. (2012b). RWRMDA: predicting novel human microRNA-disease associations. *Mol. Biosyst.* 8 2792–2798. 10.1039/c2mb25180a 22875290

[B16] Chen X. Xie D. Zhao Q. You Z. H. (2017a). MicroRNAs and complex diseases: from experimental results to computational models. *Brief Bioinform.* 10.1093/bib/bbx130 [Epub ahead of print]. 29045685

[B17] Chen X. Yan C. C. Zhang X. You Z. H. (2017b). Long non-coding RNAs and complex diseases: from experimental results to computational models. *Brief Bioinform.* 18 558–576. 10.1093/bib/bbw060 27345524 PMC5862301

[B18] Chen X. Yan G. Y. (2013). Novel human lncRNA-disease association inference based on lncRNA expression profiles. *Bioinformatics* 29 2617–2624. 10.1093/bioinformatics/btt426 24002109

[B19] Coneva V. Simopoulos C. Casaretto J. A. El-Kereamy A. Guevara D. R. Cohn J. (2014). Metabolic and co-expression network-based analyses associated with nitrate response in rice. *BMC Genomics* 15:1056. 10.1186/1471-2164-15-1056 25471115 PMC4301927

[B20] Dehury B. Behera S. K. Negi S. (2017). Overcoming the limitation of GWAS platforms using systems biology approach. *Curr. Bioinform.* 12 156–170. 10.2174/15748936116661604261708

[B21] He Y. H. Liang X. F. He S. Yuan X. C. Wang Q. C. Cai W. J. (2017). Circadian clock gene of grass carp (*Ctenopharyngodon idellus*): genomic structure and tissue expression pattern of period1 gene. *Curr. Bioinform.* 12 312–319. 10.2174/1574893611666160527101628

[B22] Holland D. O. Johnson M. E. (2018). Stoichiometric balance of protein copy numbers is measurable and functionally significant in a protein-protein interaction network for yeast endocytosis. *PLoS Comput. Biol.* 14:e1006022. 10.1371/journal.pcbi.1006022 29518071 PMC5860782

[B23] Huang R. Jiang L. Zheng J. Wang T. Wang H. Huang Y. (2013). Genetic bases of rice grain shape: so many genes, so little known. *Trends Plant Sci.* 18 218–226. 10.1016/j.tplants.2012.11.001 23218902

[B24] Jensen L. J. Kuhn M. Stark M. Chaffron S. Creevey C. Muller J. (2009). STRING 8–a global view on proteins and their functional interactions in 630 organisms. *Nucleic Acids Res.* 37 D412–D416. 10.1093/nar/gkn760 18940858 PMC2686466

[B25] Jiang J. Xing F. Zeng X. X. Zou Q. (2018). RicyerDB: a database for collecting rice yield-related genes with biological analysis int. *J. Biol. Sci.* 14 965–970. 10.7150/ijbs.23328 29989091 PMC6036756

[B26] Kohler S. Bauer S. Horn D. Robinson P. N. (2008). Walking the interactome for prioritization of candidate disease genes. *Am. J. Hum. Genet.* 82 949–958. 10.1016/j.ajhg.2008.02.013 18371930 PMC2427257

[B27] Lee I. (2011). Probabilistic functional gene societies. *Prog. Biophys. Mol. Biol.* 106 435–442. 10.1016/j.pbiomolbio.2011.01.003 21281658

[B28] Lei L. Chen J. Liu Y. Wang L. Zhao G. Chen Z. Y. (2018). Dietary wheat bran oil is equally as effective as rice bran oil in reducing plasma cholesterol. *J. Agric. Food Chem.* 66 2765–2774. 10.1021/acs.jafc.7b06093 29502409

[B29] Li F. Xie J. Zhu X. Wang X. Zhao Y. Ma X. (2018). Genetic basis underlying correlations among growth duration and yield traits revealed by GWAS in rice (*Oryza sativa* L.). *Front. Plant Sci.* 9:650. 10.3389/fpls.2018.00650 29872443 PMC5972282

[B30] Li J. Li H. Y. Zhi J. K. Shen C. Z. Yang X. S. Xu J. C. (2017). Codon usage of expansin genes in *Populus trichocarpa*. *Curr. Bioinform.* 12 452–461. 10.2174/1574893611666161008195145

[B31] Li M. Zheng R. Q. Li Q. Wang J. X. Wu F. X. Zhang Z. H. (2016). Prioritizing disease genes by using search engine algorithm. *Curr. Bioinform.* 11 195–202. 10.2174/1574893611666160125220905

[B32] Li P. Guo M. Wang C. Liu X. Zou Q. (2015). An overview of SNP interactions in genome-wide association studies. *Brief. Funct. Genomics* 14 143–155. 10.1093/bfgp/elu036 25241224

[B33] Liu E. Zeng S. Chen X. Dang X. Liang L. Wang H. (2017). Identification of putative markers linked to grain plumpness in rice (*Oryza sativa* L.) via association mapping. *BMC Genet.* 18:89. 10.1186/s12863-017-0559-6 29025391 PMC5639755

[B34] Liu Y. Zeng X. He Z. Zou Q. (2017). Inferring MicroRNA-disease associations by random walk on a heterogeneous network with multiple data sources. *Ieee Acm Trans. Comput. Biol. Bioinform.* 14 905–915. 10.1109/tcbb.2016.2550432 27076459

[B35] Liu X. Tang W. H. Zhao X. M. Chen L. (2010). A network approach to predict pathogenic genes for *Fusarium graminearum*. *PLoS One* 5:e13021. 10.1371/journal.pone.0013021 20957229 PMC2949387

[B36] Luo J. W. Liu C. C. (2017). An effective method for identifying functional modules in dynamic PPI networks. *Curr. Bioinform.* 12 66–79. 10.2174/1574893611666160831113726

[B37] Mahender A. Anandan A. Pradhan S. K. Pandit E. (2016). Rice grain nutritional traits and their enhancement using relevant genes and QTLs through advanced approaches. *Springerplus* 5:2086. 10.1186/s40064-016-3744-6 28018794 PMC5148756

[B38] Maione C. Barbosa R. M. (2018). Recent applications of multivariate data analysis methods in the authentication of rice and the most analyzed parameters: a review. *Crit. Rev. Food Sci. Nutr.* 10.1080/10408398.2018.1431763 [Epub ahead of print]. 10.1080/10408398.2018.1431763 29363991

[B39] Odilbekov F. Armoniene R. Henriksson T. Chawade A. (2018). Proximal phenotyping and machine learning methods to identify *Septoria Tritici* blotch disease symptoms in wheat. *Front. Plant Sci.* 9:685. 10.3389/fpls.2018.00685 29875788 PMC5974968

[B40] Okada S. Onogi A. Iijima K. Hori K. Iwata H. Yokoyama W. (2018). Identification of QTLs for rice grain size using a novel set of chromosomal segment substitution lines derived from Yamadanishiki in the genetic background of Koshihikari. *Breed. Sci.* 68 210–218. 10.1270/jsbbs.17112 29875604 PMC5982188

[B41] Peng W. Wang J. X. Zhang Z. Wu F. X. (2016). Applications of random walk model on biological networks. *Curr. Bioinform.* 11 211–220. 10.2174/1574893611666160223200823

[B42] Raza K. (2016). Reconstruction, topological and gene ontology enrichment analysis of cancerous gene regulatory network modules. *Curr. Bioinform.* 11 243–258. 10.2174/1574893611666160115212806

[B43] Rezadoost H. Karimi M. Jafari M. (2016). Proteomics of hot-wet and cold-dry temperaments proposed in Iranian traditional medicine: a network-based Study. *Sci. Rep.* 6:30133. 10.1038/srep30133 27452083 PMC4959000

[B44] Shim J. S. Oh N. Chung P. J. Kim Y. S. Choi Y. D. Kim J. K. (2018). Overexpression of OsNAC14 improves drought tolerance in rice. *Front. Plant Sci.* 9:310. 10.3389/fpls.2018.00310 29593766 PMC5855183

[B45] Szklarczyk D. Franceschini A. Wyder S. Forslund K. Heller D. Huerta-Cepas J. (2015). STRING v10: protein-protein interaction networks, integrated over the tree of life. *Nucleic Acids Res.* 43 D447–D452. 10.1093/nar/gku1003 25352553 PMC4383874

[B46] The Gene Ontology C. (2017). Expansion of the gene ontology knowledgebase and resources. *Nucleic Acids Res.* 45 D331–D338. 10.1093/nar/gkw1108 27899567 PMC5210579

[B47] Torres R. O. Henry A. (2018). Yield stability of selected rice breeding lines and donors across conditions of mild to moderately severe drought stress. *Field Crops Res.* 220 37–45. 10.1016/j.fcr.2016.09.011 29725159 PMC5891920

[B48] Tranchevent L. C. Ardeshirdavani A. ElShal S. Alcaide D. Aerts J. Auboeuf D. (2016). Candidate gene prioritization with endeavour. *Nucleic Acids Res.* 44 W117–W121. 10.1093/nar/gkw365 27131783 PMC4987917

[B49] Tranchevent L. C. Barriot R. Yu S. Van Vooren S. Van Loo P. Coessens B. (2008). ENDEAVOUR update: a web resource for gene prioritization in multiple species. *Nucleic Acids Res.* 36 W377–W384. 10.1093/nar/gkn325 18508807 PMC2447805

[B50] Vlaic S. Conrad T. Tokarski-Schnelle C. Gustafsson M. Dahmen U. Guthke R. (2018). ModuleDiscoverer: identification of regulatory modules in protein-protein interaction networks. *Sci. Rep.* 8:433. 10.1038/s41598-017-18370-2 29323246 PMC5764996

[B51] Wang F. Song B. X. Zhao X. Miao Y. T. Li D. Y. Zhou N. (2016). Prediction and analysis of the protein-protein interaction networks for chickens, cattle, dogs, horses and rabbits. *Curr. Bioinform.* 11 131–142. 10.2174/1574893611666151203221255

[B52] Wu T. Y. Gruissem W. Bhullar N. K. (2018). Targeting intra-cellular transport combined with efficient uptake and storage significantly increases grain iron and zinc levels in rice. *Plant Biotechnol. J.* 10.1111/pbi.12943 [Epub ahead of print]. 29734523 PMC6330537

[B53] Xiong X. Duan L. Liu L. Tu H. Yang P. Wu D. (2017). Panicle-SEG: a robust image segmentation method for rice panicles in the field based on deep learning and superpixel optimization. *Plant Methods* 13:104. 10.1186/s13007-017-0254-7 29209408 PMC5704426

[B54] Xu Y. Wang X. Ding X. Zheng X. Yang Z. Xu C. (2018). Genomic selection of agronomic traits in hybrid rice using an NCII population. *Rice* 11:32. 10.1186/s12284-018-0223-4 29748895 PMC5945574

[B55] You Z. H. Huang Z. A. Zhu Z. Yan G. Y. Li Z. W. Wen Z. (2017). PBMDA: a novel and effective path-based computational model for miRNA-disease association prediction. *PLoS Comput. Biol.* 13:e1005455. 10.1371/journal.pcbi.1005455 28339468 PMC5384769

[B56] Zeng J. Li D. Wu Y. Zou Q. Liu X. (2016). An empirical study of features fusion techniques for protein-protein interaction prediction. *Curr. Bioinform.* 11 4–12. 10.2174/1574893611666151119221435

[B57] Zhang M. Li J. Chen F. Kong Q. (2018). Unary non-structural fertilizer response model for rice crops and its field experimental verification. *Sci. Rep.* 8:2792. 10.1038/s41598-018-21163-w 29434347 PMC5809603

[B58] Zhang X. D. Zhao K. X. Yang Z. M. (2018). Identification of genomic ATP binding cassette (ABC) transporter genes and Cd-responsive ABCs in *Brassica napus*. *Gene* 664 139–151. 10.1016/j.gene.2018.04.060 29709635

[B59] Zhao G. Shi J. Liang W. Xue F. Luo Q. Zhu L. (2015). Two ATP binding cassette G transporters, rice ATP binding cassette G26 and ATP Binding cassette G15, collaboratively regulate rice male reproduction. *Plant Physiol.* 169 2064–2079. 10.1104/pp.15.00262 26392263 PMC4634043

[B60] Zhou X. Bai X. Xing Y. (2018). A rice genetic improvement boom by next generation sequencing. *Curr. Issues Mol. Biol.* 27 109–126. 10.21775/cimb.027.109 28885178

[B61] Zhu L. Su F. Xu Y. Zou Q. (2018). Network-based method for mining novel HPV infection related genes using random walk with restart algorithm. *Biochim. Biophys. Acta Mol. Basis Dis.* 1864 2376–2383. 10.1016/j.bbadis.2017.11.021 29197659

